# Modulation of *C. albicans*-Induced Immune Response in Vaginal Epithelial Cells by Garcinoic Acid

**DOI:** 10.3390/microorganisms12122455

**Published:** 2024-11-29

**Authors:** Samuele Sabbatini, Linda Zatini, Eleonora Narducci, Lucrezia Rosati, Andrea Ardizzoni, Antonella Mencacci, Mario Rende, Eva Pericolini, Francesco Galli, Desirée Bartolini, Claudia Monari

**Affiliations:** 1 Department of Medicine and Surgery, Medical Microbiology Section, University of Perugia, 06123 Perugia, Italy; nardu.eleonora@gmail.com (E.N.); antonella.mencacci@unipg.it (A.M.); claudia.monari@unipg.it (C.M.); 2 Department of Pharmaceutical Sciences, University of Perugia, 06126 Perugia, Italy; linda.zatini@studenti.unipg.it (L.Z.); francesco.galli@unipg.it (F.G.); 3 Department of Medicine and Surgery, Pharmacology Division, University of Perugia, 06132 Perugia, Italy; lucrezia.rosati@studenti.unipg.it; 4 Department of Surgical, Medical, Dental and Morphological Sciences with Interest in Transplant, Oncological and Regenerative Medicine, University of Modena and Reggio Emilia, 41125 Modena, Italy; andrea.ardizzoni@unimore.it (A.A.); eva.pericolini@unimore.it (E.P.); 5 Department of Medicine and Surgery, Section of Human, Clinical and Forensic Anatomy, University of Perugia, 60132 Perugia, Italy; mario.rende@unipg.it

**Keywords:** garcinoic acid, *C. albicans*, vaginal cells, VVC-RVVC

## Abstract

Vulvovaginal candidiasis (VVC) is a prevalent women’s infection characterized by excessive inflammation and damage of the vaginal epithelium that, in its recurrent form (RVVC), causes more than three symptomatic episodes per year, impacting nearly 8% of women globally. Current antifungal treatments alleviate symptoms but often fail to restore the inflammatory homeostasis of mucosal tissue and prevent recurrences. α-Tocopherol (α-TOH) and garcinoic acid (GA), a vitamin E metabolite, with immunomodulatory properties, were investigated for the first time in vaginal epithelial cells exposed to *C. albicans* infection to assess their effects on inflammatory signaling parameters important to restore cellular homeostasis. For this purpose, the protein kinases MKK3/6, p38 stress kinase (SAPK), and ERK1/2 were studied together with c-Fos transcription factor and IL-6, IL-1α, and IL-1β secretion in A-431 vaginal epithelial cells pre-treated with GA or with α-TOH and then infected with *C. albicans*. GA, differently from α-TOH, significantly reduced the *C. albicans*-induced activation of p38-SAPK while increasing pro-survival MAPK ERK1/2 activity. This resulted in a significant reduction in the secretion levels of the inflammatory cytokines IL-6 and IL-1α, as well as IL-1β. Overall, our data indicate that GA holds potential for restoring the immuno-metabolic properties of the vaginal epithelium exposed to *C. albicans* infection, which may help to treat inflammatory symptoms in VVC/RVVC.

## 1. Introduction

*C. albicans* is both a significant component of the human mycobiota and the primary fungal pathogen worldwide leading to a wide range of diseases, from mild mucosal infections to severe systemic candidiasis, particularly in immunocompromised patients [1]. Vulvovaginal candidiasis (VVC) is a prevalent condition, affecting about 75% of women at least once in their lifetime [2], with recurrent VVC (RVVC) being diagnosed when more than three episodes occur per year, impacting nearly 8% of women globally [3].

*C. albicans* typically resides as a commensal in the vaginal mucosa, establishing a delicate and intricate balance with host immunity and local microbial communities. Disrupting this balance can promote its overgrowth and the transition from yeast to hyphal form, which plays a crucial role in the pathogenesis of VVC [4]. Indeed, only the hyphal form has the capability to induce cell damage and activate the epithelial immune response, primarily driven by candidalysin, a cytolytic toxin secreted by *C. albicans* hyphae [5].

The recognition of a massive burden of *C. albicans* hyphae by vaginal epithelial cells (VECs) leads to the strong and sustained activation of mitogen-activated protein kinase (MAPK) pathways. This, in turn, leads to p38-mediated c-Fos activation [6] and the release of antimicrobial peptides, chemokines, and pro-inflammatory cytokines that are essential for the recruitment of innate immune cells, mainly neutrophils, promoting a chronic inflammatory state of the vaginal mucosa [4,6,7,8,9,10,11,12,13,14].

While there is growing recognition that mucosal damage is driven by an exaggerated inflammatory response, current therapeutic approaches rely solely on antifungal treatments that may provide symptoms relief but often fail to prevent recurrences. Adjuvant immunotherapies, designed to address hyper-inflammation, could potentially serve as a therapeutic option to enhance the immune response and alleviate symptoms in patients with VVC.

Plant-derived natural products have long been employed in traditional medicine. Each year, a significant number of newly discovered natural compounds necessitate systematic efforts to understand their effectiveness and roles as bioactive components or templates for drug development. A notable example in the realm of phytomedicine is the African plant *Garcinia kola* [15]. Originally recognized for its antimicrobial properties [16], other health-promoting activities have been documented for the different preparations obtained from this plant, including free radical scavenging [17], antioxidant [18], and anti-inflammatory [19] effects. The study of the multiple bioactive compounds responsible for these properties has revealed the great potential of this plant as a natural platform for drug discovery and pharmacological studies on plant metabolites. The latter include the vitamin E analogue garcinoic acid (GA) [20]. Also referred to as trans-13′-carboxy-δ-tocotrienol, GA presents a carboxylic moiety on the terminal carbon atom of the isoprenic side chain of a phenolic terpenoid, showing close structural analogy with vitamin E. GA is also formed during the hepatic and gut microbiota metabolism of dietary δ-tocotrienol (δ-T3) [20], a member of the tocotrienol family of compounds. Alongside tocopherols (TOHs), which are characterized by a phytyl side chain, these isoprenic terpenoids compose the main group of vitamin E-related molecules, with alpha-tocopherol (α-TOH) being the actual essential form [21] and the main fat-soluble antioxidant of the cell membrane. In addition, α-TOH is involved in gene regulation and detoxification mechanisms of human tissues [22], immune function modulation [23,24], skin health, and several other physiological processes, including protein kinase-mediated cytoprotection and cell cycle regulation (reviewed in [22,25,26,27]).

Recent research has demonstrated that vitamin E side-chain carboxylation significantly enhances its anti-inflammatory potential [28,29]. Consistent with this observation, GA has been observed to represent a major anti-inflammatory component in the extract of *Garcinia kola* seeds [19]. Again, recent work has shown that GA may hold therapeutic potential for reducing the cytokine storm of severe SARS-CoV-2 and other coronavirus infections [30]. Much less characterized are the biological properties of this metabolite and, more in general, of vitamin E analogues in microorganisms. To the best of our knowledge, only one study examined the anti-inflammatory effects of vitamin E in response to *C. albicans* [24]. This study demonstrated that the acetate form of this vitamin significantly downregulates pro-inflammatory cytokine pathways of human gingival fibroblasts and THP-1 mononuclear cells upon infection. At present, there are no studies on the immunomodulatory properties of GA in response to *C. albicans* infection.

Based on these premises, the aim of this in vitro study was to provide a first evaluation of GA effects on the immuno-inflammatory response of VECs to *C. albicans* infection; α-TOH was utilized as a reference compound.

## 2. Materials and Methods

### 2.1. C. albicans Strain and Growth Conditions

The reference strain *C. albicans* SC5314 (ATCC MYA-2876) was freshly cultivated from frozen glycerol stocks stored at −80 °C. Prior to each experiment, the strain was cultured in a liquid yeast extract–peptone–dextrose medium (YPD; Scharlab S.L., Barcelona, Spain) at 37 °C under aerobic conditions for 18 h. After incubation, yeast cells were washed twice with phosphate-buffered saline (PBS) and counted by using a hemocytometer. The suspension was further diluted with sterile PBS to reach the desired concentration of the inoculum.

### 2.2. Vaginal Epithelial Cell Line

The A-431 cell line from vaginal epithelial squamous cell carcinoma (ATCC CLR-1555) was cultured in Dulbecco’s Modified Eagle Medium (DMEM; Invitrogen, Carlsbad, CA, USA) supplemented with 1% penicillin/streptomycin solution (Euroclone, Milan, Italy) and 10% fetal bovine serum (FBS; Sigma-Aldrich, St. Louis, MO, USA). The cell line was maintained by subculturing it twice a week at a temperature of 37 °C and 5% CO_2_.

### 2.3. α-Tocopherol and Garcinoic Acid

α-Tocopherol (1667600) was purchased from Sigma-Aldrich (Sigma-Aldrich, St. Louis, MO, USA), and garcinoic acid (GA) was extracted from *Garcinia kola* seeds as reported in [28]. The compounds were dissolved in dimethyl sulfoxide (DMSO; vehicle), so that the stock concentration was 100 mM and the final vehicle concentration was less than 0.001% (*v/v*) in all experiments.

### 2.4. Analysis of Cell Viability (MTT Assay)

The effect of alpha-tocopherol (α-TOH; Sigma-Aldrich, St. Louis, MO, USA) or garcinoic acid (GA; isolated from *Garcinia kola* seeds as reported in [28]) on A-431 cell line viability was evaluated by using the 3-(4,5-dimethylthiazol-2-yl)-2,5-diphenyl-2H-tetrazolium bromide (MTT) colorimetric assay according to the procedure previously published in [31]. Briefly, vaginal cells were seeded at a density of 10,000 cells/well on 96-well plates. After 24 h of culture, media were removed and fresh complete media (100 µL), containing increasing concentrations of α-TOH or GA (1, 5, 10, 25, 50, and 100 µM) were added; then, cells were incubated in a humidified 5% CO_2_ atmosphere at 37 °C for 4, 24, and 48 h. Cells maintained in medium supplemented with DMSO or ethanol (EtOH) were used as controls. After treatment, the cells were incubated with complete medium containing MTT (0.5 mg/mL). After 2 h of incubation, the media were removed, and DMSO (200 µL) was added to each well to dissolve the formed formazan crystals. The absorbance at 570 nm was measured by using a microplate spectrophotometer (Tecan Infinite M200; Tecan, Männedorf, Switzerland).

### 2.5. C. albicans Morphological Analysis

For *C. albicans* morphological analysis, the A-431 cells (8 × 10^5^ cells/well) were seeded into TC-treated 6-well microtiter plates and incubated overnight at 37 °C and 5% CO_2_. Following 48 h incubation without compounds, the cells were co-incubated with *C. albicans* SC5314 (100 µL, 10 multiplicity of infection (MOI) and, 10^7^ cells/well) for 3 h under the same conditions used for protein extraction and Western blotting experiments. Morphological analysis was performed by using an inverted light microscope (Eurotek by Orma, Milan, Italy), and representative microphotographs were captured directly from the 6-well plates by using a digital camera at 200× and 400× magnifications.

### 2.6. Protein Extraction and Western Blotting

The A-431 cells (8 × 10^5^ cells/well) were incubated overnight at 37 °C and 5% CO_2_. After incubation, the cells were treated with 2 mL of complete medium containing α-TOH (25 and 50 µM) or GA (10 and 25 µM) for 48 h under the same conditions. The choice of pre-treatment for 48 h was based on cellular uptake data related to vitamin E and GA that showed maximum uptake between 48 and 72 h after treatment. After pre-treatment, the cells were co-incubated with *C. albicans* SC5314 (100 µL, 10 multiplicity of infection (MOI), and 10^7^ cells/well) for 3 h under the same conditions according to previously published papers with some modifications [4]. Then, the supernatant was removed (and stored at −80 °C), two washes were performed with sterile PBS, and cell lysis buffer (Cell Signaling Technology, Danvers, MA, USA) containing protease and phosphatase inhibitors (Thermo Fisher Scientific, Waltham, MA, USA) (100 µL in each well) was added. The cells were scraped off and kept on ice for 30 min, followed by sonication for 3 rounds in a sonicator bath (30 s and then 1 min on ice each time). Finally, after centrifugation at 14,000 rpm for 20 min at 4 °C, the supernatant containing total proteins was collected for quantification by using the bicinchoninic acid (BCA) method (Euroclone SpA, Pero, Italy). The samples were then stored at −20 °C until use.

For Western blotting, proteins (10–20 µg) were resolved via 4–12% sodium dodecyl sulphate–polyacrylamide gel electrophoresis (SDS–PAGE) minigels (Novex Wedge Well Tris-Glycine gel; Invitrogen) and then transferred to nitrocellulose membranes (Millipore, Bedford, MA, USA) for immunoblot analysis. The blocked membranes were incubated overnight at 4 °C with the primary antibodies anti-c-FOS (1:1000), anti-phospho-p38 (1:1000), anti-phospho-MKK3/6 (1:1000), anti-MKK3 (1:1000), anti-phospho-MAPK (ERK1/2) (1:1000), anti-MAPK (ERK1/2) (1:1000), anti-β-actin (1:1000), anti-GAPDH (1:2000), and anti-β-tubulin (1:1000) by Cell Signaling Technology. Membranes were washed and then incubated with the secondary antibodies anti-rabbit immunoglobulin G (IgG), horseradish peroxidase (HRP)-linked antibody (1:2000; Cell Signaling Technology), or anti-mouse IgG, HRP-linked antibody (1:10,000; Bio-Rad, Hercules, CA, USA) for 2 h at room temperature. After washing with tris-buffered saline with Tween-20 0.1% (TBST), immunoreactive signals were detected, as evidenced by the chemiluminescence reaction detected by using ECL Clarity Max (Bio-Rad, Hercules, CA, USA). The immunoreactive proteins were highlighted via a chemiluminescence reaction. The quantification of bands was performed with a ChemiDoc Imaging System, Bio-Rad, and protein levels were normalized against housekeeping proteins.

### 2.7. Enzyme-Linked Immunosorbent Assay (ELISA) for IL-6, Il-1α, and IL-1β

According to the instructions of the manufacturers, supernatant cytokines levels after 48 h of GA pre-treatment and 12 h of treatment with *C. albicans* were measured by using an IL-6 ELISA kit (ELK Biotechnology, Twin Helix, Rho, Italy), IL-1α ELISA kit, (Peprotech, Cranbury, NJ, USA), or IL-1β PicoKine ELISA kit (BosterBio, Pleasanton, CA, USA; EK0392). The levels of absorbance detected at 450 nm by a DTX880 Multimode Detector microplate reader (Beckman Coulter, Brea, CA, USA) were used to determine the levels of the cytokines by using a curve of the authentic standard.

### 2.8. Statistical Analysis

Statistical analysis was conducted by using Prism v.9.4.1 (GraphPad, San Diego, CA, USA). Statistical analysis was performed by using analysis of variance (ANOVA) or *t*-test. All data are expressed as means ± standard deviations (SDs).

## 3. Results

### 3.1. Effects of GA and α-TOH on Viability of Vaginal Epithelial Cells

**Figure 1:**
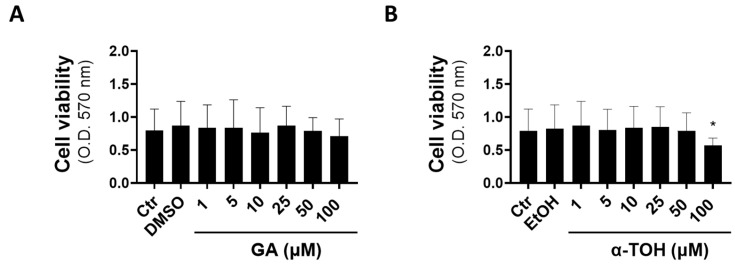
Effects of GA and α-TOH treatments on vaginal cell viability. A-431 cells were exposed to increasing levels of GA (**A**) or α-TOH (**B**) between 1 and 100 µM for 48 h and then assessed for viability by the MTT test. Data are the means ± SDs of triplicate determinations. * *p* < 0.05 (Ctr vs. all treatments). Ctr = uninfected cells.

By using the MTT assay to monitor cell viability, we found absence of toxicity for GA in the concentration range 1–100 µM in vaginal epithelial cells, whereas α-TOH showed a significant decrease in cell viability only at a 100 µM final concentration, suggesting the potential toxicity of exposure to pharmacological levels of this molecule (Figure 1).

Based on the low toxicity of both compounds in vaginal epithelial cells (Figure 1) and their concentration-dependent effects in other cell models of inflammation and toxicity [32,33,34,35], we decided to evaluate the immunomodulatory effects of GA at 10 and 25 µM and α-TOH at 25 and 50 µM.

### 3.2. Evaluation of Effects of GA and α-TOH on Candida albicans-Induced p-38 Activity in Vaginal Epithelial Cells

**Figure 2:**
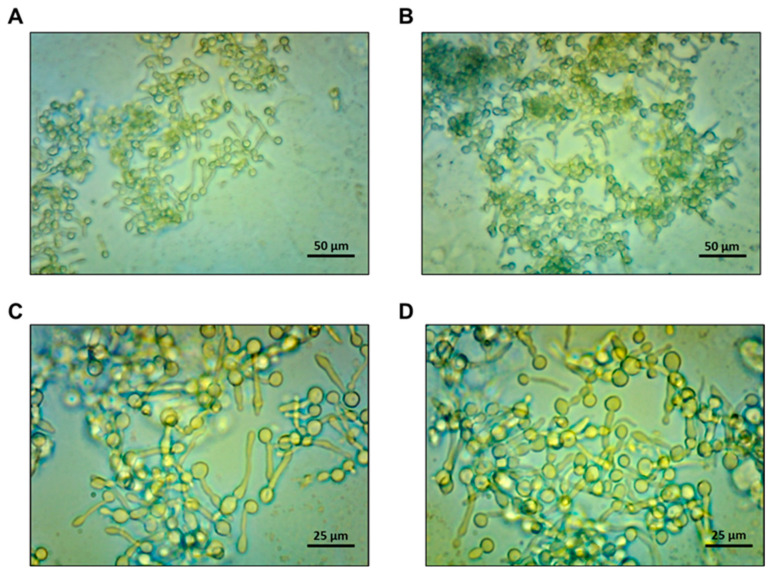
Microscopic analysis of VECs infected for 3 h with *C. albicans* SC5314. The representative images, observed by optical microscopy, show the fungal cells in the transition to the hyphal form. The pictures were taken at magnifications of 200× (**A**,**B**) and 400× (**C**,**D**).

Stress-activated protein kinases, particularly p38-SAPK, play a central role in the pro-inflammatory effects of *C. albicans* vaginal infection [4,6]. To assess the possible effect of GA and α-TOH on *C. albicans*-induced p38/c-Fos activation in VECs, we pre-treated the cells with GA (10 and 25 µM) or α-TOH (25 and 50 µM) for 48 h. This pre-treatment duration was based on previous evidence showing that the maximum uptake of these molecules and the formation of vitamin E metabolites in epithelial cells occurred between 48 and 72 h of incubation [36]. Then, given that previous results with the *C. albicans* reference strain SC5314 and A431 cells showed that over 98% of yeast cells form a germ tube after 3 h of infection [37], the cells were infected with *C. albicans* (reference strain SC5314) at the MOI of 10 for 3 h; then, we checked the morphological state of *C. albicans* cells after VEC infection. As expected, the majority of fungal cells exhibited the germ tube form (Figure 2).

**Figure 3:**
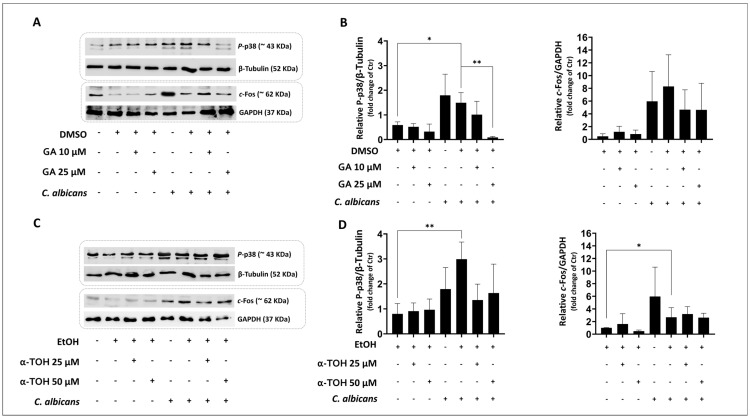
Effects of GA and α-TOH pre-treatments on phospho (P)-p38 and c-Fos levels in VECs infected with *C. albicans*. A-431 cells were pre-treated with 10 and 25 µM GA (**A**,**B**) or with 25 and 50 µM α-TOH (**C**,**D**) for 48 h and then infected for 3 h with *C. albicans*. P-p38 and c-Fos activation were determined by using Western blot analysis. The immunoblot images are representative of three independent experiments with similar results. The densitometric data depicted in the histograms are the means ± SDs of three independent experiments. * *p* < 0.05; ** *p* < 0.001 (UCV vs. *C. albicans*-infected cells); ** *p* < 0.001 (*C. albicans*-infected cells vs. *C. albicans*-infected cells + GA). Ctr = uninfected cells; UCV = uninfected cells + vehicle.

In accordance with previous results [6], *C. albicans* infection activated the p38 pathway, resulting in increased levels of P-p38 and its downstream transcriptional protein c-Fos (Figure 3A,B). Pre-treatment with 25 µM GA significantly inhibited *C. albicans*-induced p38 activation, whereas the levels of c-Fos only showed a trend toward a reduction (Figure 3B). Conversely, pre-treatment with α-TOH (25 and 50 µM) did not significantly modify the *C. albicans*-induced activation of both p38 and c-Fos protein expression (Figure 3C,D). Based on these results, in subsequent experiments, we focused on the potential immunomodulatory activity of GA.

**Figure 4:**
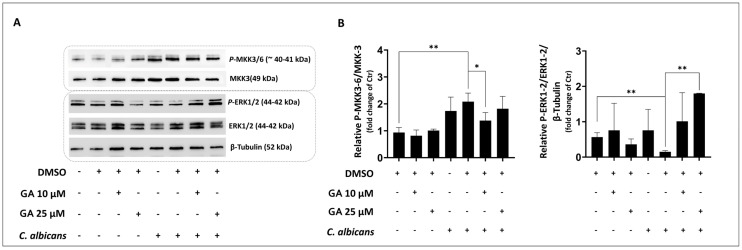
Effects of GA pre-treatment on phospho (P)-MKK3/6 and P-ERK1/2 levels of VECs infected with *C. albicans*. A-431 cells were pre-treated with 10 and 25 µM GA for 48 h and then exposed for 3 h to *C. albicans*. P-MKK3/6 and P-ERK1/2 activation was determined by using Western blot analysis. The immunoblot images are representative of three independent experiments with similar results (**A**). The densitometric data in the histograms depict the means ± SDs of three independent experiments (**B**). ** *p* < 0.001 (UCV vs. *C. albicans*-infected cells); * *p* < 0.05; ** *p* < 0.001 (*C. albicans*-infected cells vs. *C. albicans*-infected cells + GA). Ctr = uninfected cells; UCV = uninfected cells + vehicle.

Given that it is well known that p38 activation is strongly regulated by MAPK kinases MKK3/6 [38] and MAPK-ERK1/2 [39], Western blotting experiments were performed to evaluate GA ability to modulate these pathways. Our results show that *C. albicans* induced significant expression of P-MKK3/6; such expression was significantly reduced by pre-treatment with 10 µM GA (Figure 4A,B).

Moreover, *C. albicans* infection did not trigger the activation of P-ERK1/2. Pre-treatment with 25 µM GA significantly increased P-ERK1/2 expression in infected VECs (Figure 4A,B).

Altogether, these results suggest that GA may have the potential to modulate key protein kinases associated with the immune response of VECs to *C. albicans* infection, namely, the stress-activated p38 and pro-survival MAPK-ERK [39].

### 3.3. Garcinoic Acid Pre-Treatment Reduced IL-6, IL-1α, and IL-1β Secretion in Vaginal Epithelial Cells Exposed to C. albicans Infection

IL-6, IL-1α, and IL-1β are three key pro-inflammatory cytokines involved in the immunopathogenesis of VVC, and their production is induced by exposure to *C. albicans* in hyphal form [13], as well as candidalysin [40,41]. In this context, signal transduction and transcriptional effects of the p38 pathway influence the expression of these cytokines with an important role in the immuno-metabolic response to pathogens. Therefore, their secretion levels were measured to assess the immunomodulatory properties of GA pre-treatment in VECs infected for 12 h with *C. albicans* at the MOI of 10. Our results show that the 48 h pre-treatment with 25 µM GA significantly reduced the secretion of all the three cytokines in the *C. albicans*-infected VECs (Table 1).

## 4. Discussion

Vulvovaginal candidiasis is primarily affected by the local innate immune response, with VECs being identified as the main cellular target and the key inflammatory player of this infectious disease. Current models of VVC etiopathogenesis suggest that the presence of a significant load of filamentous forms and the production of candidalysin [40] significantly contribute to the onset of the non-protective inflammatory response characteristic of this infection [1,4,6,12]. Common antifungal treatments primarily focus on alleviating symptoms and eradicating the pathogen but often fail to restore the inflammatory homeostasis of mucosal tissues, leading to recurrent infections.

In this in vitro study, we assessed for the first time the potential of α-TOH and the vitamin E metabolite GA in modulating the epithelial immune response to *C. albicans* infection. The role of innate immunity in the onset of vaginal infections is well documented [1], and reducing inflammation in VVC may represent an important strategy to restore the homeostatic conditions of VECs, ancillary to antifungal therapy. Stress-activated protein kinases, particularly p38-SAPK, appear to play a central role in the pro-inflammatory effects of *C. albicans* vaginal infection. Accordingly, the exposure of vaginal (as well as oral) epithelial cells to high burdens of *C. albicans* hyphae or to candidalysin, strongly induces p38-SAPK signaling and the activity of its downstream transcription factor c-Fos, thus driving the cell toward a pro-inflammatory phenotype [4,6,13,42]. Based on these premises, we explored whether GA, compared with its analogue α-TOH, could reduce these molecular and cellular hallmarks of *C. albicans* infection in the vaginal epithelial cell line A-431. Here, we show that *C. albicans* is able to activate p38 and c-Fos in these cells and that GA pre-treatment (25 µM), differently from its vitamin analogue α-TOH, prevents p38 activation.

MAPK cascade activation is a complex and interconnected process involved in the innate immunity of epithelial cells and its changes during *C. albicans* infection. In this respect, the extracellular signal-regulated kinases 1 and 2 (ERK1/2) pathway appears to play a central role, with signaling activation demonstrated post-infection in both oral [42] and vaginal epithelial cells [4].

However, we observed reduced activity of this kinase in our experimental model, possibly due to the different time of infection (3 h in our study vs. 2 h post-infection reported in [4]). Our findings align more closely with recent results obtained by Zhang et al. [13], which suggest that ERK1/2 phosphorylation, in VECs, could be specific to the yeast rather than the hyphal form of the infection.

Interestingly, GA (25 µM) was able to modulate the crosstalk between p38-SAPK and MAPK ERK1/2, which is important to control essential cellular processes of the infected cells, including innate immunity and inflammatory gene expression. In this respect, GA may have the potential to mitigate the immune response to *C. albicans* infection in VECs.

To further explore the effects of GA on the VECs’ immune response to *C. albicans*, we assessed the levels of IL-6, IL-1α, and IL-1β, the pro-inflammatory cytokines produced in response to *C. albicans* hyphae or to candidalysin through the activation of different signal transduction pathways that include p38 SAPK [4,13,40]. As expected, *C. albicans* has a proinflammatory effect also on VECs, by inducing an increase in their levels. GA has been demonstrated to prevent this effect, which is in agreement with the p38 pathway modulation response to this vitamin E-related metabolite. This important finding, once again, demonstrates the potential of GA as a natural agent and modulator of the innate immunity response to the host, studied in different experimental models, including exposure to bacterial components, such as LPS [19], and SARS-CoV-2 infection [30].

It is important to emphasize that these findings represent a promising starting point for investigating the immunomodulatory effects of GA in the context of *C. albicans* infection and its restoring effects on the vaginal epithelium. The limitation of this study is the use of an immortalized cell line which may not accurately reflect the physiological responses of human epithelial cells. Further experiments using primary vaginal cells may strengthen these results, paving the way for more complex experimental settings.

Overall, our data show that GA pre-treatment is able to reduce the immune response of VECs to *C. albicans* infection by balancing the ERK1/2-MAPK and p38-SAPK pathways.

The effects of GA on other players in VVC may include the inhibition of inflammatory pathways in resident monocyte–macrophages and infiltrating neutrophils; in fact, these inflammatory cells have been reported in the literature to respond to the anti-inflammatory activity of this and other vitamin E metabolites [43], which is worth investigating in future studies to better characterize the therapeutic potential of this metabolite in VVC.

## 5. Conclusions

Vaginal candidiasis is considered an immunopathological condition, given that the symptoms are primarily driven by local inflammation. Although most episodes of recurrent VVC caused by *C. albicans* respond well to azole therapy, long-term antifungal maintenance regimens are required. GA, by modulating the immune response to *C. albicans* infection, could help alleviate symptoms. This approach is especially valuable, as prolonged antifungal use can lead to the appearance of drug-resistant strains and cause unwanted side effects.

Hence, this in vitro study provided the first evidence of the immunomodulatory and anti-inflammatory potential of GA in the context of *C. albicans* vaginal infection. Our findings indicate that GA can modulate the MKK3/6-p38/SAPK pathway, activated by *C. albicans* in VECs, through the activation of pro-survival MAPK ERK1/2.

**Figure 5:**
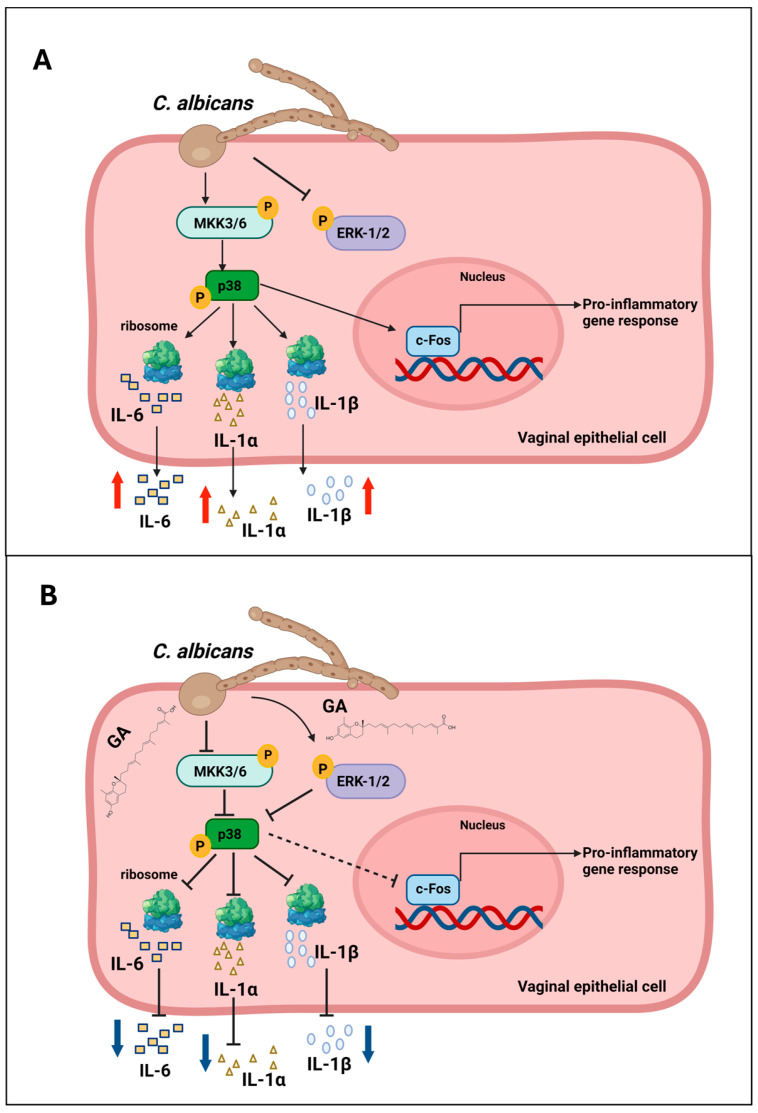
Proposed model of mechanistic aspects of GA immuno-modulatory effects in vaginal epithelial cells exposed to *C. albicans* infection. The MKK3/6-p38/SAPK pathway and its regulatory interplay with pro-survival MAPK ERK1/2 are molecular targets of GA, with modulating activity on IL-6, IL-1 α, and IL-1β levels secreted by the infected vaginal epithelium. The effect of GA on these cytokines may preserve the immune-metabolic properties of the vaginal epithelium and support post-infection recovery and the restoration of cellular homeostasis. (**A**) Mechanism of action of *C. albicans* on vaginal epithelial cells. (**B**) Effect of pre-treatment with GA on vaginal cells infected with *C. albicans.* The dotted line in the arrows indicates that there is a trend toward a non-significant reduction in the proposed event; the solid line indicates a significant reduction/inhibition; red arrows indicate increased production; blue arrows indicate decreased secretion. The image was produced by using BioRender software (https://www.biorender.com; license number: C81D6A59-0002).

This modulation is associated with a significant reduction in the secretion of three key pro-inflammatory cytokines, IL-6, IL-1α, and IL-1β, which are typically upregulated during *C. albicans* vaginal infection and contribute to the inflammatory milieu (Figure 5).

Our findings pave the way to other levels of investigation on this and other long-chain metabolites of vitamin E in VVC/RVVC. In this context, GA deserves further investigation as a possible co-adjuvant of anti-fungal therapy to lessen *C. albicans*-induced pro-inflammatory activation and to help restore epithelial homeostasis after fungal infection. Finally, further in vivo pharmacokinetic studies will be essential to determining whether 25 µM is feasible or if adjustments are needed for practical application.

## Figures and Tables

**Table 1:** Effects of GA pre-treatment on IL-6, IL-1α, and IL-1β levels of *C. albicans*-infected VECs.

pg/mL Mean ± SD	UCV	GA (25 μM)	*C. albicans*	*C. albicans* + GA (25 μM)
IL-6	18.19 ± 2.46	20.10 ± 1.43	55.19 ± 16.30	28.17 ± 1.61 **
IL-1α	85.58 ± 2.79	81.15 ± 4.35	90.23 ± 6.09	80.66 ± 4.56 *
IL-1β	4.71 ± 1.80	3.81 ± 0.40	27.03 ± 2.41	13.10 ± 2.96 ****

A-431 cells, pre-treated or not with GA (25 µM) for 48 h, were infected with *C. albicans* (at the MOI of 10). IL-6, IL-1α, and IL-1β levels were measured by specific ELISA kits in cell supernatants. Data are expressed as the means ± SDs of 3 different experiments. **** *p* < 0.0001, ** *p* < 0.01, and * *p* < 0.05 (*C. albicans* vs. *C. albicans* + GA). UCV = uninfected cells + vehicle.

## Data Availability

The raw data supporting the conclusions of this article will be made available by the authors on request.
